# Supplementary material for: Differential increase of hippocampal subfield volume after socio-affective mental training relates to reductions in diurnal cortisol
Source: eLife. 2024 Aug 28;12:RP87634. doi: 10.7554/eLife.87634 (PMC11357357; doi:10.7554/eLife.87634)
Supplement: Supplementary file 1. — (a) Descriptive statistics T0-T1. (b) Descriptive statistics T1-T3. (c) T0-T1 change statistics. (d) T1-T3 change statistics. (e) T1-T3 change statistics – Training cohort 1 and 2 Affect versus Perspective. (f) T1-T2 change. (g) T2-T3 change. (h) Subfield-specific changes following the Training Modules, controlling for the other two ipsilateral subfields. (i) Overall change in subfield volume. (j) Sex differences (female versus male) in hippocampal subfield volumes. (k) Descriptive statistics mean subfield functional network change T0-T1. (l) Descriptive statistics mean subfield functional network change T1-T3. (m) Functional connectivity network change T0-T1. (n) Functional connectivity network change T1-T3. (o) Functional connectivity network change T1-T3: Training cohort 1 and 2 Affect versus Perspective. (p) Functional connectivity network change T1-T2. (q) Functional connectivity network change T2-T3. (r) Correlating change in subfield volume and diurnal cortisol indices in Affect. (s) Association between stress-markers and within functional network sub-regions in Affect and Perspective. (t) Correlating change in subfield functional network and diurnal cortisol indices in Affect. (u) Correlating change in subfield volume and diurnal cortisol indices in Presence. (v) Correlating change in subfield volume and diurnal cortisol indices in Perspective. (w) Correlating change in subfield function and diurnal cortisol indices in Presence. (x) Correlating change in subfield function and diurnal cortisol indices in Perspective. (y) Overall effects of cortisol markers on hippocampal volume in Presence, Affect, and Perspective. (z) Overall effects of cortisol markers on hippocampal function in Presence, Affect, and Perspective. (za) Effects of hair cortisol markers on hippocampal subfield volume in Presence, Affect, and Perspective. (zb) Effects of hair cortisol markers on hippocampal subfield function in Presence, Affect, and Perspective. [file elife-87634-supp1.docx]

**Supplementary File 1a**. Descriptive statistics T0-T1, p-values are uncorrected, q values reported when p<.05.

| Presence | LSUB | LCA1-3 | LCA4/DG | RSUB | RCA1-3 | RCA4/DG |
| --- | --- | --- | --- | --- | --- | --- |
| *t-values* | 1,335 | 0,836 | -0,452 | -0,623 | 0,788 | -0,751 |
| *p-value* | 0,183 | 0,404 | 0,652 | 0,534 | 0,432 | 0,454 |
| *Mean* | 4,616 | 30,930 | -4,686 | -7,279 | 13,244 | -2,419 |
| *std* | 83,141 | 205,390 | 51,976 | 98,493 | 166,340 | 64,548 |
| *CI min* | -13,209 | -13,105 | -15,830 | -28,396 | -22,419 | -16,258 |
| *CI max* | 22,442 | 74,965 | 6,458 | 13,838 | 48,907 | 11,421 |
| Affect TC3 | |  |  |  |  |  |
| *t-values* | 1,195 | 1,456 | 1,739 | 1,528 | 1,334 | 0,060 |
| *p-value* | 0,234 | 0,147 | 0,084 | 0,128 | 0,184 | 0,952 |
| *Mean* | 4,295 | 46,443 | 7,312 | 19,426 | 23,344 | 3,705 |
| *std* | 90,258 | 206,540 | 43,280 | 121,990 | 187,470 | 63,729 |
| *CI min* | -18,821 | -6,454 | -3,773 | -11,816 | -24,670 | -12,617 |
| *CI max* | 27,411 | 99,340 | 18,396 | 50,669 | 71,358 | 20,027 |
| RCC |  |  |  |  |  |  |
| *t-values* | -0,955 | -0,497 | -1,698 | -0,126 | -2,526 | 0,778 |
| *p-value* | 0,341 | 0,620 | 0,091 | 0,900 | 0,012, q=0.072 | 0,437 |
| *Mean* | -14,000 | 6,259 | -12,111 | -0,870 | -50,259 | 10,407 |
| *std* | 66,176 | 156,220 | 41,762 | 91,759 | 139,760 | 79,943 |
| *CI min* | -32,063 | -36,381 | -23,510 | -25,916 | -88,406 | -11,413 |
| *CI max* | 4,063 | 48,900 | -0,712 | 24,175 | -12,112 | 32,228 |

**Supplementary File 1b**. Descriptive statistics T1-T3, p-values are uncorrected, q values reported when p<.05.

| Perspective | LSUB | LCA1-3 | LCA4/DG | RSUB | RCA1-3 | RCA4/DG |
| --- | --- | --- | --- | --- | --- | --- |
| *t-values* | 0,456 | -1,143 | -0,463 | 0,573 | -2,118 | 1,291 |
| *p-value* | 0,649 | 0,254 | 0,644 | 0,567 | 0,035, q>0.1 | 0,198 |
| *Mean* | 4,434 | -23,048 | -1,398 | 2,108 | -39,602 | 12,024 |
| *std* | 71,215 | 137,810 | 45,224 | 99,892 | 208,470 | 76,355 |
| *CI min* | -11,116 | -53,139 | -11,273 | -19,704 | -85,122 | -4,649 |
| *CI max* | 19,984 | 7,043 | 8,477 | 23,921 | 5,917 | 28,697 |
| Affect |  |  |  |  |  |  |
| *t-values* | 1,121 | 2,495 | 0,235 | 0,210 | 2,374 | 0,394 |
| *p-value* | 0,263 | 0,013, q=0.078 | 0,814 | 0,833 | 0,018, q>0.1 | 0,694 |
| *Mean* | 8,424 | 25,511 | 1,489 | -2,098 | 40,120 | 5,087 |
| *std* | 63,328 | 130,470 | 36,293 | 112,520 | 181,300 | 76,124 |
| *CI min* | -4,691 | -1,509 | -6,027 | -25,399 | 2,573 | -10,678 |
| *CI max* | 21,539 | 52,531 | 9,005 | 21,204 | 77,666 | 20,852 |
| RCC |  |  |  |  |  |  |
| *t-values* | -1,102 | -1,118 | -1,052 | -0,409 | 1,052 | -0,557 |
| *p-value* | 0,271 | 0,264 | 0,294 | 0,683 | 0,294 | 0,578 |
| *Mean* | -6,864 | -21,845 | -3,700 | -8,591 | 15,673 | -1,827 |
| *std* | 75,284 | 137,630 | 45,632 | 104,680 | 155,170 | 71,276 |
| *CI min* | -21,090 | -47,853 | -12,323 | -28,372 | -13,650 | -15,297 |
| *CI max* | 7,363 | 4,162 | 4,923 | 11,190 | 44,995 | 11,642 |

**Supplementary File 1c**. T0-T1 change statistics, p-values are uncorrected, q values reported when p<.05.

| Affect TC3 vs Presence | LSUB | LCA1-3 | LCA4/DG | RSUB | RCA1-3 | RCA4/DG |
| --- | --- | --- | --- | --- | --- | --- |
| *t-value* | -0,065 | 0,454 | 1,519 | 1,487 | 0,401 | 0,548 |
| *p-value* | 0,948 | 0,650 | 0,130 | 0,139 | 0,689 | 0,584 |
| *Cohens D* | -0,009 | 0,065 | 0,217 | 0,212 | 0,057 | 0,078 |
| Affect TC3 vs RCC |  |  |  |  |  |  |
| *t-value* | 1,359 | 1,228 | 2,175 | 1,036 | 2,452 | -0,461 |
| *p-value* | 0,176 | 0,221 | 0,031, q>0.1 | 0,302 | 0,015, q=0.09 | 0,645 |
| *Cohens D* | 0,194 | 0,175 | 0,311 | 0,148 | 0,350 | -0,066 |
| Presence vs RCC |  |  |  |  |  |  |
| *t-value* | 1,522 | 0,883 | 0,875 | -0,317 | 2,248 | -1,021 |
| *p-value* | 0,130 | 0,379 | 0,383 | 0,752 | 0,026, q>0.1 | 0,308 |
| *Cohens D* | 0,217 | 0,126 | 0,125 | -0,045 | 0,321 | -0,146 |

**Supplementary File 1d**. T1-T3 change statistics, p-values are uncorrected, q values reported when p<.05.

| Affect vs Perspective | LSUB | LCA1-3 | LCA4/DG | RSUB | RCA1-3 | RCA4/DG |
| --- | --- | --- | --- | --- | --- | --- |
| *t-value* | 0,417 | 2,360 | 0,458 | -0,245 | 2,930 | -0,604 |
| *p-value* | 0,677 | 0,019,  q>0.1 | 0,647 | 0,807 | 0,004, q=0.022 | 0,547 |
| *Cohens D* | 0,050 | 0,282 | 0,055 | -0,029 | 0,350 | -0,072 |
| Affect vs RCC |  |  |  |  |  |  |
| *t-value* | 1,504 | 2,460 | 0,861 | 0,417 | 0,935 | 0,641 |
| *p-value* | 0,134 | 0,014,  q=0.084 | 0,390 | 0,677 | 0,351 | 0,522 |
| *Cohens D* | 0,180 | 0,294 | 0,103 | 0,050 | 0,112 | 0,077 |
| Perspective vs RCC |  |  |  |  |  |  |
| *t-value* | 1,025 | -0,067 | 0,359 | 0,659 | -2,139 | 1,250 |
| *p-value* | 0,306 | 0,947 | 0,720 | 0,510 | 0,033,  q>0.1 | 0,212 |
| *Cohens D* | 0,123 | -0,008 | 0,043 | 0,079 | -0,256 | 0,149 |

**Supplementary File 1e**. T1-T3 change statistics – Training cohort 1 and 2 *Affect* versus *Perspective.* P-values are uncorrected, q values reported when p<.05.

| TC1 | LSUB | LCA1-3 | LCA4/DG | RSUB | RCA1-3 | RCA4/DG |
| --- | --- | --- | --- | --- | --- | --- |
| *t-value* | 1,557 | 2,549 | 1,263 | 0,214 | 4,243 | -0,224 |
| *p-value* | 0,122 | 0,012, q=0.072 | 0,209 | 0,831 | 0,000  q<0.001 | 0,823 |
| *Cohens D* | 0,273 | 0,447 | 0,222 | 0,038 | 0,744 | -0,039 |
| TC2 |  |  |  |  |  |  |
| *t-value* | -0,819 | 0,447 | -0,300 | -0,757 | 0,102 | -0,681 |
| *p-value* | 0,414 | 0,656 | 0,765 | 0,451 | 0,919 | 0,497 |
| *Cohens D* | -0,149 | 0,081 | -0,055 | -0,138 | 0,019 | -0,124 |

**Supplementary File 1f**. T1-T2 change. P-values are uncorrected, q values reported when p<.05.

| T1-T2  Affect vs Perspective | LSUB | LCA1-3 | LCA4/DG | RSUB | RCA1-3 | RCA4/DG |
| --- | --- | --- | --- | --- | --- | --- |
| *t-value* | 0,612 | 1,751 | 0,944 | 0,404 | 3,743 | -0,174 |
| *p-value* | 0,541 | 0,082 | 0,347 | 0,687 | 0,000  q<0.001 | 0,862 |
| *Cohens D* | 0,108 | 0,308 | 0,166 | 0,071 | 0,659 | -0,031 |
| Affect vs RCC |  |  |  |  |  |  |
| *t-value* | 1,995 | 0,967 | 0,582 | 0,974 | 2,637 | 0,622 |
| *p-value* | 0,048  q>0.1 | 0,335 | 0,562 | 0,332 | 0,009  q=0.054 | 0,535 |
| *Cohens D* | 0,351 | 0,170 | 0,102 | 0,171 | 0,464 | 0,110 |
| Perspective vs RCC |  |  |  |  |  |  |
| *t-value* | 1,271 | -0,938 | -0,448 | 0,508 | -1,454 | 0,786 |
| *p-value* | 0,206 | 0,350 | 0,655 | 0,613 | 0,148 | 0,433 |
| *Cohens D* | 0,224 | -0,165 | -0,079 | 0,089 | -0,256 | 0,138 |

**Supplementary File 1g**. T2-T3 change. P-values are uncorrected, q values reported when p<.05.

| T2-T3  Affect vs Perspective | LSUB | LCA1-3 | LCA4/DG | RSUB | RCA1-3 | RCA4/DG |
| --- | --- | --- | --- | --- | --- | --- |
| *t-value* | -0,033 | 1,768 | -0,176 | -0,582 | 0,641 | -0,748 |
| *p-value* | 0,974 | 0,079 | 0,860 | 0,561 | 0,523 | 0,456 |
| *Cohens D* | -0,005 | 0,293 | -0,029 | -0,096 | 0,106 | -0,124 |
| Affect vs RCC |  |  |  |  |  |  |
| *t-value* | 0,212 | 2,691 | 0,832 | -0,302 | -1,006 | 0,326 |
| *p-value* | 0,832 | 0,008,  q=0.048 | 0,407 | 0,763 | 0,316 | 0,745 |
| *Cohens D* | 0,035 | 0,445 | 0,138 | -0,050 | -0,167 | 0,054 |
| Perspective vs RCC |  |  |  |  |  |  |
| *t-value* | 0,239 | 0,828 | 0,988 | 0,296 | -1,628 | 1,075 |
| *p-value* | 0,811 | 0,409 | 0,325 | 0,768 | 0,106 | 0,284 |
| *Cohens D* | 0,040 | 0,137 | 0,164 | 0,049 | -0,269 | 0,178 |

**Supplementary File 1h.** Subfield-specific changes following the Training Modules, controlling for the other two ipsilateral subfields

|  | LSUB | LCA1-3 | LCA4/DG | RSUB | RCA1-3 | RCA4/DG |
| --- | --- | --- | --- | --- | --- | --- |
| *Presence vs Active Control* | t=0.475  p>0.1  D=0.069 | t=-0.033  p>0.1  D=-0.004 | t=-1.514  p>0.1  D=-0.216 | t=-1.365  p>0.1  D=-0.195 | t=-0.162  p>0.1  D=-0.023 | t=-0.210  p>0.1  D=-0.03 |
| *Affect vs Perspective* | t=0.793  p>0.1  D=-0.03 | t=2.298, p=0.022  D=0.275 | t=0.924  p>0.1  D=-0.012 | t=0.883  p>0.1  D=-0.018 | t=3.045  p=0.0025  Q=0.015  D=0.364 | t=-0.978  P>0.1  D=-0.117 |

**Supplementary File 1i**. Overall change in subfield volume.

| Training vs RCC | LSUB | LCA1-3 | LCA4/DG | RSUB | RCA1-3 | RCA4/DG |
| --- | --- | --- | --- | --- | --- | --- |
| *t-value* | 1,033 | 0,702 | 1,035 | -1,348 | 0,774 | 0,017 |
| *p-value* | 0,303 | 0,484 | 0,302 | 0,180 | 0,440 | 0,986 |
| *Cohens D* | 0,172 | 0,117 | 0,172 | -0,225 | 0,129 | 0,003 |

**Supplementary File 1j**. Sex differences (female versus male) in hippocampal subfield volumes.

|  | LSUB | LCA1-3 | LCA4/DG | RSUB | RCA1-3 | RCA4/DG |
| --- | --- | --- | --- | --- | --- | --- |
| baseline | t=2.423  p=0.008  q=0.049 | t=2.061  p=0.021  q=0.121 | t=3.671  p<0.001  q=0.001 | t=-0.520  p=0.698  q>0.05 | t=3.570  p<0.001  q>0.002 | t=2.555  p=0.006  q=0.034 |
| baseline+ ICVcontrol | t=1.739  p=0.042  q>0.1 | t=-0.171  p>0.1  q>0.1 | t=1.259  p>0.1  q>0.1 | t=-0.358  p>0.1  q>0.1 | t=1.962  p=0.025  q>0.1 | t=1.201  p>0.1  q>0.1 |
| *Main change analysis* |  |  |  |  |  |  |
| *Affect* versus *Perspective*  ICVcontrol | t=0.514  p>0.05  q>0.05 | t=2.588  p=0.01  q=0.06 | t=0.677  p>0.05  q>0.05 | t=-0.262  p>0.05  q>0.05 | t=3.065  p=0.002  q=0.014 | t=-0.511  p>0.05  q>0.05 |
| *Presence* versus *Active Control*  ICVcontrol | t=-0.101  p>0.05  q>0.05 | t=0.214  p>0.05  q>0.05 | t=1.236  p>0.05  q>0.05 | t=1.462  p>0.05  q>0.05 | t=0.256  p>0.05  q>0.05 | t=0.406  p>0.05  q>0.05 |

**Supplementary File 1k**. Descriptive statistics mean subfield functional network change T0-T1. Main focus was on CA1-3 based on volumetric observations and are highlighted in bold. For these multiple comparisons (FDRq, corrected for two subfields) values are reported if uncorrected p values are below p<.05.

| Presence | LSUB | LCA1-3 | LCA4/DG | RSUB | RCA1-3 | RCA4/DG |
| --- | --- | --- | --- | --- | --- | --- |
| *t-values* | -1,223 | **-0,803** | -0,915 | -0,757 | **-0,507** | 0,387 |
| *p-value* | 0,223 | **0,423** | 0,361 | 0,450 | **0,613** | 0,699 |
| *Mean* | -0,004 | **-0,001** | -0,008 | 0,000 | **0,009** | 0,004 |
| *std* | 0,091 | **0,089** | 0,093 | 0,089 | **0,099** | 0,087 |
| *CI min* | -0,023 | **-0,020** | -0,028 | -0,018 | **-0,012** | -0,014 |
| *CI max* | 0,015 | **0,018** | 0,011 | 0,019 | **0,029** | 0,023 |
| Affect TC3 |  |  |  |  |  |  |
| *t-values* | -1,089 | **-0,247** | -0,105 | -1,053 | **-1,071** | 0,587 |
| *p-value* | 0,278 | **0,805** | 0,917 | 0,294 | **0,285** | 0,558 |
| *Mean* | -0,004 | **0,003** | 0,000 | -0,004 | **0,002** | 0,007 |
| *std* | 0,078 | **0,091** | 0,082 | 0,085 | **0,081** | 0,096 |
| *CI min* | -0,024 | **-0,021** | -0,021 | -0,026 | **-0,019** | -0,018 |
| *CI max* | 0,016 | **0,026** | 0,021 | 0,018 | **0,022** | 0,032 |
| RCC |  |  |  |  |  |  |
| *t-values* | -0,521 | **-1,462** | 0,023 | 0,198 | **1,384** | 0,296 |
| *p-value* | 0,603 | **0,145** | 0,981 | 0,843 | **0,168** | 0,767 |
| *Mean* | -0,001 | **-0,011** | 0,000 | 0,009 | **0,029** | 0,004 |
| *std* | 0,107 | **0,095** | 0,100 | 0,094 | **0,097** | 0,080 |
| *CI min* | -0,030 | **-0,036** | -0,028 | -0,017 | **0,002** | -0,017 |
| *CI max* | 0,028 | **0,015** | 0,027 | 0,034 | **0,055** | 0,026 |

**Supplementary File 1l**. Descriptive statistics mean subfield functional network change T1-T3. Main focus was on CA1-3 based on volumetric observations and are highlighted in bold. For these multiple comparisons (FDRq, corrected for two subfields) values are reported if uncorrected p values are below p<.05.

| Perspective | LSUB | LCA1-3 | LCA4/DG | RSUB | RCA1-3 | RCA4/DG |
| --- | --- | --- | --- | --- | --- | --- |
| *t-value* | 0,046 | **-0,071** | 0,461 | -1,443 | **-2,012** | -1,089 |
| *p-value* | 0,963 | **0,943** | 0,645 | 0,150 | **0,045,**  **q>0.1** | 0,277 |
| *Mean* | -0,003 | **-0,007** | 0,003 | -0,012 | **-0,024** | -0,006 |
| *std* | 0,088 | **0,100** | 0,092 | 0,089 | **0,081** | 0,099 |
| *CI min* | -0,022 | **-0,029** | -0,017 | -0,032 | **-0,041** | -0,028 |
| *CI max* | 0,017 | **0,015** | 0,023 | 0,007 | **-0,006** | 0,016 |
| Affect |  |  |  |  |  |  |
| *t-values* | 1,050 | **0,139** | 0,899 | 1,116 | **1,691** | 0,569 |
| *p-value* | 0,295 | **0,889** | 0,369 | 0,265 | **0,092** | 0,570 |
| *Mean* | 0,007 | **-0,005** | 0,007 | 0,011 | **0,010** | 0,010 |
| *std* | 0,103 | **0,110** | 0,100 | 0,092 | **0,098** | 0,098 |
| *CI min* | -0,015 | **-0,028** | -0,013 | -0,008 | **-0,010** | -0,010 |
| *CI max* | 0,028 | **0,018** | 0,028 | 0,030 | **0,031** | 0,030 |
| RCC |  |  |  |  |  |  |
| *t-values* | 0,225 | **1,206** | -0,608 | -0,839 | **-0,701** | -0,436 |
| *p-value* | 0,822 | **0,229** | 0,544 | 0,402 | **0,484** | 0,663 |
| *Mean* | 0,000 | **0,005** | -0,005 | -0,006 | **-0,010** | 0,001 |
| *std* | 0,079 | **0,079** | 0,071 | 0,089 | **0,092** | 0,092 |
| *CI min* | -0,015 | **-0,010** | -0,019 | -0,023 | **-0,028** | -0,017 |
| *CI max* | 0,014 | **0,020** | 0,008 | 0,011 | **0,007** | 0,018 |

**Supplementary File 1m**. Functional connectivity network change T0-T1. Main focus was on CA1-3 based on volumetric observations and are highlighted in bold. For these multiple comparisons (FDRq, corrected for two subfields) values are reported if uncorrected p values are below p<.05.

| Affect TC3 vs Presence | |  |  |  |  |  |
| --- | --- | --- | --- | --- | --- | --- |
|  | **LSUB** | **LCA1-3** | **LCA4/DG** | **RSUB** | **RCA1-3** | **RCA4/DG** |
| *t-value* | 0,058 | **0,366** | 0,541 | -0,230 | **-0,411** | 0,151 |
| *p-value* | 0,953 | **0,715** | 0,589 | 0,818 | **0,682** | 0,880 |
| *Cohens D* | 0,008 | **0,052** | 0,077 | -0,033 | **-0,058** | 0,021 |
| Affect TC3 vs RCC | |  |  |  |  |  |
| *t-value* | -0,347 | **0,782** | -0,080 | -0,785 | **-1,556** | 0,177 |
| *p-value* | 0,729 | **0,435** | 0,936 | 0,433 | **0,121** | 0,860 |
| *Cohens D* | -0,049 | **0,111** | -0,011 | -0,112 | **-0,221** | 0,025 |
| Presence vs RCC | |  |  |  |  |  |
| *t-value* | -0,430 | **0,491** | -0,607 | -0,626 | **-1,283** | 0,046 |
| *p-value* | 0,668 | **0,624** | 0,545 | 0,532 | **0,201** | 0,964 |
| *Cohens D* | -0,061 | **0,070** | -0,086 | -0,089 | **-0,182** | 0,006 |

**Supplementary File 1n**. Functional connectivity network change T1-T3. Main focus was on CA1-3 based on volumetric observations and are highlighted in bold. For these multiple comparisons (FDRq, corrected for two subfields) values are reported if uncorrected p values are below p<.05.

| Affect vs Perspective | |  |  |  |  |  |
| --- | --- | --- | --- | --- | --- | --- |
|  | **LSUB** | **LCA1-3** | **LCA4/DG** | **RSUB** | **RCA1-3** | **RCA4/DG** |
| *t-value* | 0,644 | **0,137** | 0,272 | 1,674 | **2,420** | 1,088 |
| *p-value* | 0,520 | **0,891** | 0,786 | 0,095 | **0,016**  **q=0.032** | 0,278 |
| *Cohens D* | 0,077 | **0,016** | 0,032 | 0,200 | **0,289** | 0,130 |
| Affect vs RCC |  |  |  |  |  |  |
| *t-value* | 0,575 | **-0,703** | 1,023 | 1,326 | **1,631** | 0,681 |
| *p-value* | 0,566 | **0,483** | 0,307 | 0,186 | **0,104** | 0,496 |
| *Cohens D* | -0,049 | **0,111** | -0,011 | -0,112 | **-0,221** | 0,025 |
| Perspective vs RCC | |  |  |  |  |  |
| *t-value* | -0,113 | **-0,824** | 0,709 | -0,458 | **-0,939** | -0,472 |
| *p-value* | 0,910 | **0,410** | 0,479 | 0,648 | **0,348** | 0,637 |
| *Cohens D* | -0,061 | **0,070** | -0,086 | -0,089 | **-0,182** | 0,006 |

**Supplementary File 1o**. Functional connectivity network change T1-T3: Training cohort 1 and 2 *Affect* versus *Perspective.* Main focus was on CA1-3 based on volumetric observations and are highlighted in bold. For these multiple comparisons (FDRq, corrected for two subfields) values are reported if uncorrected p values are below p<.05. Explorative analyses take all 6 subfields into account.

| TC1 | LSUB | LCA1-3 | LCA4/DG | RSUB | RCA1-3 | RCA4/DG |
| --- | --- | --- | --- | --- | --- | --- |
| *t-value* | -0,362 | **-0,985** | -0,339 | -0,254 | **-0,417** | -0,085 |
| *p-value* | 0,718 | **0,326** | 0,735 | 0,800 | **0,677** | 0,933 |
| *Cohens D* | -0,063 | **-0,172** | -0,059 | -0,044 | **-0,073** | -0,015 |
| TC2 |  |  |  |  |  |  |
| *t-value* | 1,189 | **1,171** | 0,669 | 2,873 | **3,815** | 1,683 |
| *p-value* | 0,237 | **0,244** | 0,505 | 0,005  q=0.03 (6) | **0,000**  **q<0.001** | 0,095 |
| *Cohens D* | 0,215 | **0,212** | 0,121 | 0,520 | **0,691** | 0,305 |

**Supplementary File 1p**. Functional connectivity network change T1-T2. Main focus was on CA1-3 based on volumetric observations and are highlighted in bold. For these multiple comparisons (FDRq, corrected for two subfields) values are reported if uncorrected p values are below p<.05.

| Affect vs Perspective |  |  |  |  |  |  |
| --- | --- | --- | --- | --- | --- | --- |
|  | **LSUB** | **LCA1-3** | **LCA4/DG** | **RSUB** | **RCA1-3** | **RCA4/DG** |
| *t-value* | -0,423 | **0,055** | -0,713 | 0,810 | **2,121** | 0,737 |
| *p-value* | 0,673 | **0,956** | 0,477 | 0,419 | **0,036**  **q=0.072** | 0,463 |
| *Cohens D* | -0,074 | **0,010** | -0,126 | 0,143 | **0,373** | 0,130 |
| Affect vs RCC |  |  |  |  |  |  |
| *t-value* | -0,766 | **-1,978** | -0,285 | 0,088 | **0,923** | -0,061 |
| *p-value* | 0,445 | **0,051** | 0,776 | 0,930 | **0,358** | 0,952 |
| *Cohens D* | -0,049 | **0,111** | -0,011 | -0,112 | **-0,221** | 0,025 |
| Perspective vs RCC |  |  |  |  |  |  |
| *t-value* | -0,287 | **-1,967** | 0,487 | -0,780 | **-1,375** | -0,846 |
| *p-value* | 0,775 | **0,052** | 0,627 | 0,437 | **0,172** | 0,399 |
| *Cohens D* | -0,061 | **0,070** | -0,086 | -0,089 | **-0,182** | 0,006 |

**Supplementary File 1q**. Functional connectivity network change T2-T3. Main focus was on CA1-3 based on volumetric observations and are highlighted in bold. For these multiple comparisons (FDRq, corrected for two subfields) values are reported if uncorrected p values are below p<.05.

| Affect vs Perspective | LSUB | LCA1-3 | LCA4/DG | RSUB | RCA1-3 | RCA4/DG |
| --- | --- | --- | --- | --- | --- | --- |
| *t-value* | 1,378 | **0,202** | 1,037 | 1,678 | **1,501** | 0,911 |
| *p-value* | 0,170 | **0,840** | 0,301 | 0,096 | **0,136** | 0,364 |
| *Cohens D* | 0,227 | **0,033** | 0,171 | 0,277 | **0,248** | 0,150 |
| Affect vs RCC |  |  |  |  |  |  |
| *t-value* | 1,488 | **0,760** | 1,598 | 1,630 | **1,192** | 0,996 |
| *p-value* | 0,139 | **0,449** | 0,112 | 0,105 | **0,235** | 0,321 |
| *Cohens D* | -0,049 | **0,111** | -0,011 | -0,112 | **-0,221** | 0,025 |
| Perspective vs RCC |  |  |  |  |  |  |
| *t-value* | 0,045 | **0,533** | 0,499 | -0,121 | **-0,367** | 0,043 |
| *p-value* | 0,964 | **0,595** | 0,619 | 0,904 | **0,714** | 0,966 |
| *Cohens D* | -0,061 | **0,070** | -0,086 | -0,089 | **-0,182** | 0,006 |

**Supplementary File 1r. Correlating change in subfield volume and diurnal cortisol indices in *Affect*.** Main focus was on CA1-3 based on volumetric observations and are highlighted in bold. For these multiple comparisons (FDRq, corrected for two subfields) values are reported if uncorrected p values are below p<.05.

|  | LSUB | LCA1-3 | LCA4/DG | RSUB | RCA1-3 | RCA4/DG |
| --- | --- | --- | --- | --- | --- | --- |
| CAR | 1,007, p>0.1 | **-0,355, p>0.1** | 0,166, p>0.1 | -1,364, p>0.1 | **-1,543, p>0.1** | -0,404, p>0.1 |
| Slope | -0,283, p>0.1 | **-0,878, p>0.1** | 0,728, p>0.1 | 0,634, p>0.1 | **-1,245, p>0.1** | -1,716, p<0.1 |
| AUC_g_ | -0,945, p>0.1 | **-2,237, p=0.028, q=0.056** | 0,636, p>0.1 | -0,222, p>0.1 | **-2,283, p=0.025, q=0.05** | -1,446, p>0.1 |

**Supplementary File 1s. Association between stress-markers and within functional network sub-regions in *Affect* and *Perspective*.**

| *Affect* | LCA1-3 - PI | RCA1-3 - mPFC | *Perspective* | LCA1-3 - PI | RCA1-3 - mPFC |
| --- | --- | --- | --- | --- | --- |
| CAR | -0.939, p>0.1 | -0.137, p>0.1 |  | -0.202, p>0.1 | -0.217, p>0.1 |
| Slope | 0.652, p>0.1 | 0.385, p>0.1 |  | 1.385, p>0.1 | 0.660, p>0.1 |
| AUC_g_ | -0.625, p>0.1 | -0.484, p>0.1 |  | -0.411,p>0.1 | 0.003, p>0.1 |

**Supplementary File 1t. Correlating change in subfield functional network and diurnal cortisol indices in *Affect*.** Main focus was on CA1-3 based on volumetric observations and are highlighted in bold. For these multiple comparisons (FDRq, corrected for two subfields) values are reported if uncorrected p values are below p<.05.

|  | LSUB | LCA1-3 | LCA4/DG | RSUB | RCA1-3 | RCA4/DG |
| --- | --- | --- | --- | --- | --- | --- |
| CAR | 0,066, p>0.1 | **-0,476, p>0.1** | -0,535, p>0.1 | -0,764, p>0.1 | **-0,425, p>0.1** | -0,534, p>0.1 |
| Slope | 0,800, p>0.1 | **2,653, p=0.009,**  **q=0.018** | 1,662, p>0.1 | 1,385, p>0.1 | **0,773, p>0.1** | 1,102, p>0.1 |
| AUC_g_ | 0,914, p>0.1 | **2,261, p=0.026,**  **q=0.052** | 1,638, p>0.1 | -0,697, p>0.1 | **0,024, p>0.1** | -0,447, p>0.1 |

**Supplementary File 1u. Correlating change in subfield volume and diurnal cortisol indices in *Presence*.** Main focus was on CA1-3 based on volumetric observations and are highlighted in bold. For these multiple comparisons (FDRq, corrected for two subfields) values are reported if uncorrected p values are below p<.05.

|  | LSUB | LCA1-3 | LCA4/DG | RSUB | RCA1-3 | RCA4/DG |
| --- | --- | --- | --- | --- | --- | --- |
| CAR | 1,520,  p>0.1 | **1,484,**  **p>0.1** | 1,908,  p=0.06 | 1,391,  p>0.1 | **0,241,**  **p>0.1** | 0,891,  p>0.1 |
| Slope | -1,052  p>0.1 | **-1,777,**  **p=0.08** | -2,890,  p=0.005, q=0.03 | -1,408,  p>0.1 | **-1,736,**  **p=0.086** | -0,451,  p>0.1 |
| AUC_g_ | 0,023  p>0.1 | **-0,356,**  **p>0.1** | -0,546,  p>0.1 | -0,223,  p>0.1 | **-0,118,**  **p>0.1** | 1,116,  p>0.1 |

**Supplementary File 1v. Correlating change in subfield volume and diurnal cortisol indices in *Perspective*.** Main focus was on CA1-3 based on volumetric observations and are highlighted in bold. For these multiple comparisons (FDRq, corrected for two subfields) values are reported if uncorrected p values are below p<.05.

|  | LSUB | LCA1-3 | LCA4/DG | RSUB | RCA1-3 | RCA4/DG |
| --- | --- | --- | --- | --- | --- | --- |
| CAR | 0,394, p>0.1 | **0,575, p>0.1** | 0,916, p>0.1 | 1,535, p>0.1 | **0,868, p>0.1** | 2,263, p=0.026,  q>0.1 |
| Slope | -0,388, p>0.1 | **-1,409, p>0.1** | -0,904, p>0.1 | 0,825, p>0.1 | **-1,152, p>0.1** | -0,690, p>0.1 |
| AUC_g_ | -1,136, p>0.1 | **-1,060, p>0.1** | -0,018, p>0.1 | 0,995, p>0.1 | **-0,838, p>0.1** | -0,727, p>0.1 |

**Supplementary File 1w. Correlating change in subfield function and diurnal cortisol indices in *Presence*.** Main focus was on CA1-3 based on volumetric observations and are highlighted in bold. For these multiple comparisons (FDRq, corrected for two subfields) values are reported if uncorrected p values are below p<.05.

|  | LSUB | LCA1-3 | LCA4/DG | RSUB | RCA1-3 | RCA4/DG |
| --- | --- | --- | --- | --- | --- | --- |
| CAR | 1,250,  p>0.1 | **1,133,**  **p>0.1** | 0,608,  p>0.1 | 0,877,  p>0.1 | **-0,090,**  **p>0.1** | 0,160,  p>0.1 |
| Slope | 0,637,  p>0.1 | **1,103,**  **p>0.1** | 1,187,  p>0.1 | 0,096,  p>0.1 | **0,424,**  **p>0.1** | -0,243,  p>0.1 |
| AUC_g_ | 0,514,  p>0.1 | **1,102,**  **p>0.1** | 0,546,  p>0.1 | -0,130,  p>0.1 | **-0,644,**  **p>0.1** | -1,007,  p>0.1 |

**Supplementary File 1x. Correlating change in subfield function and diurnal cortisol indices in *Perspective*.** Main focus was on CA1-3 based on volumetric observations and are highlighted in bold. For these multiple comparisons (FDRq, corrected for two subfields) values are reported if uncorrected p values are below p<.05.

|  | LSUB | LCA1-3 | LCA4/DG | RSUB | RCA1-3 | RCA4/DG |
| --- | --- | --- | --- | --- | --- | --- |
| CAR | -2,324,  p=0.023,  q>0.1 | **-1,685,**  **p=0.096** | -2,215, p=0.03,  q>0.1 | -1,464,  p>0.1 | **0,492,**  **p>0.1** | -1,148,  p>0.1 |
| Slope | 0,318,  p>0.1 | **1,027,**  **p>0.1** | -0,096,  p>0.1 | 1,912,  p=0.06 | **0,556,**  **p>0.1** | 1,283,  p>0.1 |
| AUC_g_ | -1,990,  p=0.05 | **-0,609,**  **p>0.1** | -1,678,  p>0.1 | -0,748,  p>0.1 | **-0,473,**  **p>0.1** | -0,135,  p>0.1 |

**Supplementary File 1y**. Overall effects of cortisol markers on hippocampal volume in *Presence*, *Affect*, and *Perspective*. Main focus was on CA1-3 based on volumetric observations and are highlighted in bold. For these multiple comparisons (FDRq, corrected for two subfields) values are reported if uncorrected p values are below p<.05.

|  | LSUB | LCA1-3 | LCA4/DG | RSUB | RCA1-3 | RCA4/DG |
| --- | --- | --- | --- | --- | --- | --- |
| CAR | 1,260, p>0.1 | **0,491, p>0.1** | 1,882, p<0.1 | 0,487, p>0.1 | **-1,116, p>0.1** | 1,383, p>0.1 |
| Slope | -0,561, p>0.1 | **-1,861, p<0.1** | -1,836, p<0.1 | 0,667, p>0.1 | **-1,788, p<0.1** | -1,361, p>0.1 |
| AUC_g_ | -1,383, p>0.1 | **-2,008, p<0.05**  **q>0.1** | -0,117, p>0.1 | 0,113, p>0.1 | **-2,117, p<0.03,**  **q=0.06** | -0,872, p>0.1 |

**Supplementary File 1z**. Overall effects of cortisol markers on hippocampal function in *Presence*, *Affect*, and *Perspective*. Main focus was on CA1-3 based on volumetric observations and are highlighted in bold. For these multiple comparisons (FDRq, corrected for two subfields) values are reported if uncorrected p values are below p<.05.

|  | LSUB | LCA1-3 | LCA4/DG | RSUB | RCA1-3 | RCA4/DG |
| --- | --- | --- | --- | --- | --- | --- |
| CAR | -0,666, p>0.1 | **-0,896, p>0.1** | -1,221, p>0.1 | -1,173, p>0.1 | **-0,290, p>0.1** | -1,131, p>0.1 |
| Slope | 1,416, p>0.1 | **3,024, p<0.001,**  **q=0.002** | 1,949, p<0.1 | 1,984, p<0.05  q>0.1 | **0,991, p>0.1** | 1,284, p>0.1 |
| AUC_g_ | -0,232, p>0.1 | **1,614, p>0.1** | 0,405, p>0.1 | -0,919, p>0.1 | **-0,463, p>0.1** | -0,787, p>0.1 |

**Supplementary File 1za**. Effects of hair cortisol markers on hippocampal subfield volume in *Presence*, *Affect*, and *Perspective.* Main focus was on CA1-3 based on volumetric observations and are highlighted in bold. For these multiple comparisons (FDRq, corrected for two subfields) values are reported if uncorrected p values are below p<.05. (Cortisol (HC) and Cortisone (HE)).

|  | LSUB | LCA1-3 | LCA4/DG | RSUB | RCA1-3 | RCA4/DG | |
| --- | --- | --- | --- | --- | --- | --- | --- |
| HC | -0,595, p>0.1 | **-2,574, p=0.011, q=0.022** | -0,750, p>0.1 | -1,251, p>0.1 | **-0,199, p>0.1** | | -3,138, p=0.002  q=0.012 |
| HE | -0,127, p>0.1 | **-0,040, p>0.1** | -0,204, p>0.1 | -1,765, p<0.1 | **-0,589, p>0.1** | | -0,311, p>0.1 |

**Supplementary File 1zb**. Effects of hair cortisol markers on hippocampal subfield function in *Presence*, *Affect*, and *Perspective.* Main focus was on CA1-3 based on volumetric observations and are highlighted in bold. For these multiple comparisons (FDRq, corrected for two subfields) values are reported if uncorrected p values are below p<.05. (Cortisol (HC) and Cortisone (HE)). (Cortisol (HC) and cortisone (HE)).

|  | LSUB | LCA1-3 | LCA4/DG | RSUB | RCA1-3 | RCA4/DG |
| --- | --- | --- | --- | --- | --- | --- |
| HC | -2,890, p=0.005,  q=0.03 | **-2,700, p=0.008**  **q=0.013** | -1,675, p>0.1 | -0,638, p>0.1 | **-0,019, p>0.1** | -0,329, p>0.1 |
| HE | -0,627, p>0.1 | **0,237, p>0.1** | -0,791, p>0.1 | 0,983, p<0.1 | **0,916, p>0.1** | 0,798, p>0.1 |
